# cfDNA methylome profiling for detection and subtyping of small cell lung cancers

**DOI:** 10.1038/s43018-022-00415-9

**Published:** 2022-08-08

**Authors:** Francesca Chemi, Simon P. Pearce, Alexandra Clipson, Steven M. Hill, Alicia-Marie Conway, Sophie A. Richardson, Katarzyna Kamieniecka, Rebecca Caeser, Daniel J. White, Sumitra Mohan, Victoria Foy, Kathryn L. Simpson, Melanie Galvin, Kristopher K. Frese, Lynsey Priest, Jacklynn Egger, Alastair Kerr, Pierre P. Massion, John T. Poirier, Gerard Brady, Fiona Blackhall, Dominic G. Rothwell, Charles M. Rudin, Caroline Dive

**Affiliations:** 1 Nucleic Acid Biomarker Team, Cancer Biomarker Centre, Cancer Research UK Manchester Institute, University of Manchester, Alderley Edge, UK; 2 Bioinformatics and Biostatistics Team, Cancer Biomarker Centre, Cancer Research UK Manchester Institute, University of Manchester, Alderley Edge, UK; 3 The Christie NHS Foundation Trust, Manchester, UK; 4 Department of Medicine, Memorial Sloan Kettering Cancer Center, New York, NY USA; 5 Preclinical and Pharmacology Team, Cancer Biomarker Centre, Cancer Research UK Manchester Institute, University of Manchester, Alderley Edge, UK; 6 Division of Cancer Sciences, Faculty of Biology Medicine and Health, University of Manchester, Manchester, UK; 7 Division of Allergy, Pulmonary and Critical Care Medicine, Vanderbilt University Medical Center, Nashville, TN USA; 8 Perlmutter Cancer Center, New York University Langone Health, New York, NY USA

**Keywords:** Tumour biomarkers, Small-cell lung cancer, Cancer, Cancer genomics

## Abstract

Small cell lung cancer (SCLC) is characterized by morphologic, epigenetic and transcriptomic heterogeneity. Subtypes based upon predominant transcription factor expression have been defined that, in mouse models and cell lines, exhibit potential differential therapeutic vulnerabilities, with epigenetically distinct SCLC subtypes also described. The clinical relevance of these subtypes is unclear, due in part to challenges in obtaining tumor biopsies for reliable profiling. Here we describe a robust workflow for genome-wide DNA methylation profiling applied to both patient-derived models and to patients’ circulating cell-free DNA (cfDNA). Tumor-specific methylation patterns were readily detected in cfDNA samples from patients with SCLC and were correlated with survival outcomes. cfDNA methylation also discriminated between the transcription factor SCLC subtypes, a precedent for a liquid biopsy cfDNA-methylation approach to molecularly subtype SCLC. Our data reveal the potential clinical utility of cfDNA methylation profiling as a universally applicable liquid biopsy approach for the sensitive detection, monitoring and molecular subtyping of patients with SCLC.

## Abstract (web summary)

Dive and colleagues develop an approach for genome-wide cell-free DNA methylation profiling, which can sensitively detect small cell lung cancer in liquid biopsy of patient samples, including stage I disease and molecularly defined SCLC subtypes.

## Main

SCLC represents 10–15% of lung cancer cases; it is strongly associated with tobacco smoking and characterized by high proliferation rate and early, rapid metastatic spread^1^. Most patients with SCLC present with extensive-stage (ES-SCLC) metastatic disease (stage IV); a third are diagnosed with limited-stage disease (LS-SCLC, stage IA–IIIB). SCLC is initially exceptionally responsive to platinum-based chemotherapy, although acquired resistance emerges rapidly and few patients survive beyond 1–2 years^2^. The recent addition of immunotherapy to standard chemotherapy yielded durable responses in a small subset of, as yet, undefined patients^3^. New therapeutic strategies guided by biomarkers for patient stratification are clearly needed to improve SCLC survival.

Although SCLC is treated as a homogenous disease, recent studies revealed morphologic^4^ and transcriptomic heterogeneity with several subtypes identified based on predominant transcription factor (TF) expression^1,5–7^. Preclinical studies suggest that these SCLC subtypes exhibit dynamic plasticity and have differential therapeutic vulnerabilities^8–10^ although the clinical relevance of these molecular subtypes remains obscure and obtaining tissue biopsies even at a single time point with adequate quality for transcriptional molecular analysis remains a significant challenge^11^.

We previously reported that tumor genomic alterations (copy number aberrations (CNAs) and somatic mutations) are readily detected in circulating cfDNA extracted from blood of patients with SCLC, highlighting the potential of this liquid biopsy as a tumor surrogate^12^; however, genomic profiling has not mapped to the TF-based SCLC subtypes described above^5^. DNA methylation is considered an important regulator of SCLC biology^13^ and analysis of SCLC primary tumor samples revealed epigenetically distinct subgroups^14^, though differential methylomes have not been explored in cfDNA.

Here, we describe a robust workflow for genome-wide DNA methylation profiling applied to both patient-derived models and to patient-derived cfDNA samples. These data nominate cfDNA methylation profiling as a non-invasive, sensitive and universally applicable approach to stage I–IV SCLC detection, disease monitoring and predominant subtyping. This rapid turnaround, blood-based subtyping methodology has the potential to substantially inform and accelerate future drug development in SCLC: (1) by permitting evaluation of differential response to new agents across subtypes of disease; (2) by facilitating analyses of plasticity and interconversion between subtypes as a mechanism of acquired resistance; and ultimately (3) by allowing rapid and safe enrollment of candidates to biomarker-guided clinical trials in patients with select subtypes of SCLC.

## Results

### Shared methylation patterns in preclinical models and cfDNA

**Fig. 1:**
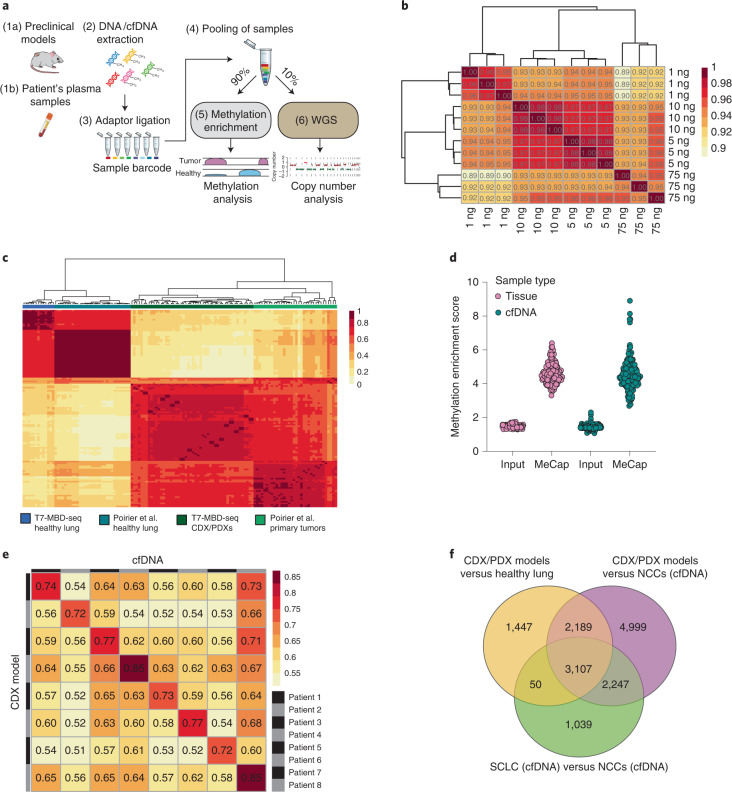
A workflow for accurate detection of SCLC methylation patterns in CDX/PDX models and cfDNA samples. **a**, T7-MBD-seq workflow. Fragmented genomic DNA or cfDNA were subjected to barcoding and pooling followed by incubation with a methyl-binding domain 2 protein (MBD2). A tenth of the pooled sample was kept as input control. NGS libraries were generated for both methylated enriched and input fractions with the aim of obtaining methylation and copy number profiles from the same original barcoded and pooled sample. Blood tube image was obtained from BioRender.com. WGS, whole-genome sequencing. **b**, Hierarchical clustering heat map showing Pearson correlation between the normalized reads per million values across the whole genome (8,956,617 300-bp windows), for varying starting amounts of DNA (in triplicate) of the lung cancer cell line H1975. **c**, Hierarchical clustering heat map of Spearman correlation of differentially methylated CpG probes (20,578 CpG probes corresponding to 14,887 300-bp windows) previously detected between healthy lung tissue (*n* = 31 individuals) and primary SCLC tumors^14^ (*n* = 34 patients) mapped to our SCLC dataset (*n* = 50 CDX and PDX models from 33 patients, *β*-values were averaged over up to three independent mice for each model) and to healthy lung samples (*n* = 13 individuals) processed through T7-MBD-seq protocol. **d**, Dot plot showing methylation enrichment scores of input control samples and methylation captured samples (MeCap) for both tissue samples (*n* = 110) and cfDNA samples (*n* = 157). **e**, Spearman correlation between methylation profiles for CDX models and cfDNA samples from the same patients (using 76,225 300-bp regions with nrpm < 1 in NCCs, but ≥1 in a CDX/PDX sample). **f**, Venn diagram showing the overlap of most significant DMRs for three different comparisons: CDX/PDXs (*n* = 50 models, as in **c**) versus healthy lung (*n* = 13 individuals) (DMRs = 6,793, |Δ*β*| ≥ 0.5, false discovery rate (FDR) ≤ 0.001), CDX/PDXs (*n* = 50 models) versus NCCs (*n* = 79 individuals) (DMRs = 12,542, |Δ*β*| ≥ 0.5, FDR ≤ 0.001) and SCLC cfDNA (*n* = 78 patients) versus NCCs (*n* = 79 individuals) (DMRs = 6,443, |Δ*β*| ≥ 0.3, FDR ≤ 0.001). Source data

**Extended Data Fig. 1:**
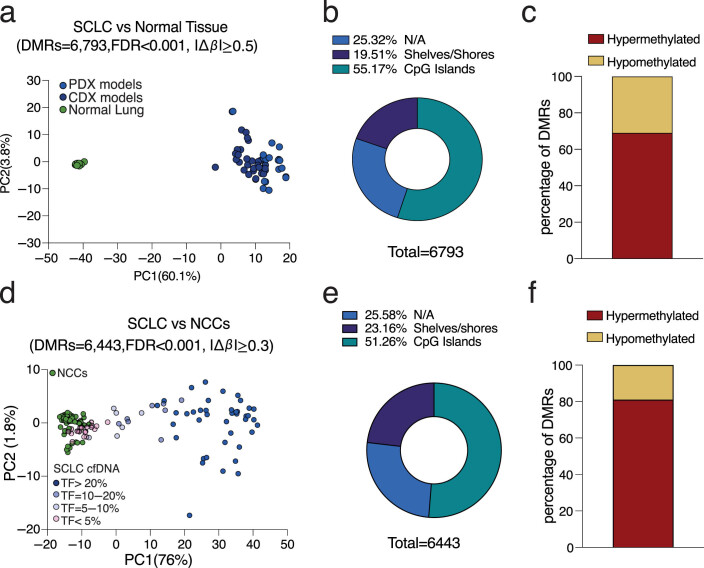
SCLC methylation patterns in preclinical models and cfDNA samples. **a**, PCA plot of CDX/PDX models (*n* = 50 models from 33 patients, *β*-values were averaged over up to three independent mice for each model) and normal lung tissue samples (*n* = 13 individuals), from PCA applied to *β*-values for the 6,793 most significant DMRs detected between CDX/PDX and normal lung. **b**, Distribution of the 6,793 DMRs over regulatory regions (CpG Islands, shores and shelves) in CDX/PDX vs normal lung comparison. **c**, Bar plot showing the percentage of the 6,793 DMRs detected as hypermethylated and hypomethylated in CDX/PDX vs normal lung comparison. **d**, PCA plot of SCLC cfDNA (*n* = 78 patients) and NCC cfDNA (*n* = 79 individuals), from PCA applied to *β*-values for the 6,443 most significant DMRs detected between SCLC cfDNA and NCC cfDNA. **e**, Distribution of the 6,443 DMRs over regulatory regions (CpG Islands, shores and shelves) in SCLC cfDNA versus NCC cfDNA comparison. **f**, Bar plot showing the percentage of the 6,443 DMRs detected as hypermethylated and hypomethylated in SCLC cfDNA versus NCC cfDNA comparison. Source data

To evaluate SCLC genome-wide DNA methylation patterns, we employed a bisulfite-free, enrichment-based next-generation sequencing (NGS) approach that incorporated an in-house library preparation method to allow sample multiplexing before enrichment (T7-MBD-seq) (Fig. 1a), which we demonstrated gave reproducible methylation profiles for DNA inputs as low as 1 ng (Fig. 1b). We initially tested this approach on DNA from 110 tissue samples; 97 from patient-derived xenografts (PDXs) or circulating tumor cell-derived explant (CDX) samples (from 50 preclinical models derived from 33 unique patients) and 13 samples of healthy lungs (Supplementary Table 1). Principal-component analysis (PCA) of the most significant differentially methylated regions (DMRs) between SCLC models and healthy controls showed distinct separation (Extended Data Fig. 1a). Consistent with previous reports, SCLC samples presented with more variable DNA methylation patterns compared to healthy lung, suggesting underlying epigenetic heterogeneity (Extended Data Fig. 1a). Overall, approximately 75% of DMRs mapped to CpG islands (CGIs), shores or shelves (Extended Data Fig. 1b). The majority of DMRs identified were hypermethylated in the tumor (69%), which is a likely consequence of using a methylation capture approach that favors high enrichment of CpG dense regions^15^ (Extended Data Fig. 1c). Methylation profiles from CDX/PDX and healthy lung tissue correlated with previously described methylation patterns from SCLC primary tumor^14^ and healthy lung profiled on the Illumina Human Methylation 450k platform (Fig. 1c), confirming the discriminatory power of the T7-MBD-seq methodology.

We next applied our T7-MBD-seq approach to a total of 157 cfDNA samples; 78 from patients with SCLC (29 LS-SCLC and 49 ES-SCLC) and 79 noncancer controls (NCCs; 45 risk- and age-matched, 26 age-matched only and 8 unmatched) (Supplementary Tables 2 and 3). Despite the lower DNA input used for cfDNA samples (range 1.83–34.4 ng) compared to CDX/PDX samples (50 ng), methylation enrichment scores were comparable across all samples (Fig. 1d). PCA analysis of the most significant DMRs between SCLC and NCC cfDNA samples segregated the majority of SCLC from NCC cfDNA samples, with the level of separation dependent on tumor fraction (Extended Data Fig. 1d). A similar breakdown of genomic regions featuring a DMR as observed in preclinical models was also seen in cfDNA samples (Extended Data Fig. 1e,f). Tissue methylation profiles of eight SCLC CDX models were compared to a corresponding cfDNA sample collected at baseline from the same donor patient. For six of eight patients bloods were collected at the same time (baseline) to derive the CDX model and assess cfDNA, for two of eight the CDX models were derived from bloods collected at disease progression. In all cases cfDNA and tissue methylation profiles were highly concordant (Fig. 1e). In addition, recurrent SCLC-specific methylation patterns observed across 50 CDX/PDX models were recapitulated across 78 SCLC cfDNA samples in which 84% (5,404 of 6,443) of DMRs detected in cfDNA were also found in the CDX/PDX tumors (Fig. 1f). Collectively, these data suggest that our T7-MBD-seq approach provides reproducible and characteristic SCLC methylation profiles in tissue, which are also readily detected in cfDNA, prompting us to extend our research efforts on cfDNA methylation as a potential biomarker for clinical application in patients with SCLC.

### A classifier for detection of SCLC from cfDNA methylation profiling

**Fig. 2:**
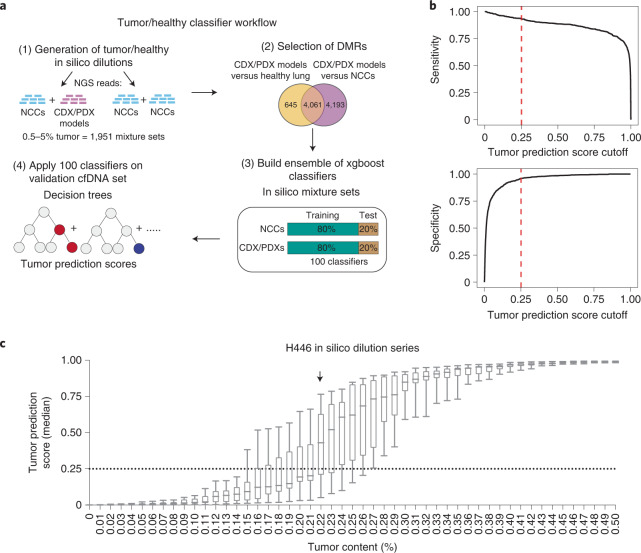
Generation of a DNA methylation classifier for sensitive tumor detection. **a**, Analysis workflow for the generation of the tumor/healthy classifier. **b**, Sensitivity and specificity metrics plotted against cutoff values for the median tumor prediction score output by the tumor/healthy classifier applied to held-out synthetic mixture sets (total of *n* = 1,951 mixture sets). Dotted lines indicate the cutoff value (0.25) that optimizes the balanced accuracy metric (average of sensitivity and specificity). **c**, Box plots of median tumor prediction scores from applying the tumor/healthy classifier to in silico serial dilutions of a fragmented SCLC cell line (H446) mixed with an NCC cfDNA sample, with varying proportions of the cell line in the mixture (*x* axis). For each proportion, 11 independent in silico dilution experiments were carried out. Boxes mark the 25th percentile (bottom), median (central bar) and 75th percentile (top); whiskers extend to minimum and maximum points. Dotted line indicates the cutoff for the tumor/healthy classifier derived as above. Arrow indicates the lowest dilution of H466 with a median value (across the 11 in silico experiments) above this cutoff (0.22% tumor content). Source data

We initially explored the extent to which DNA methylation profiling could provide a sensitive approach for blood-based detection of disease in patient samples. We applied a machine-learning approach in which a tumor/healthy classifier was trained using 4,061 DMRs found between CDX/PDX models and either healthy lung samples or a training subset of 38 of our NCC cfDNA samples (Fig. 2a and Supplementary Table 4). To replicate the lower tumor fraction often seen in cfDNA, we generated 1,951 in silico spike-in samples consisting of reads from NCC samples mixed with reads from a single CDX/PDX model (0.5–5% CDX/PDX reads). An ensemble classifier was built by repeating the training procedure 100 times, using 80% of the samples each time. Sensitivity and specificity analysis identified an optimal cutoff (0.25) for dichotomizing the median SCLC prediction score output by the ensemble classifier (Fig. 2b; Methods). In addition, we estimated the limit of detection of our approach by applying the ensemble classifier across an in silico serial dilution of the H446 SCLC cell line into a NCC cfDNA sample. Applying the cutoff value of 0.25 enabled detection of SCLC signal down to 0.22% cancer cell content (Fig. 2c).

**Fig. 3:**
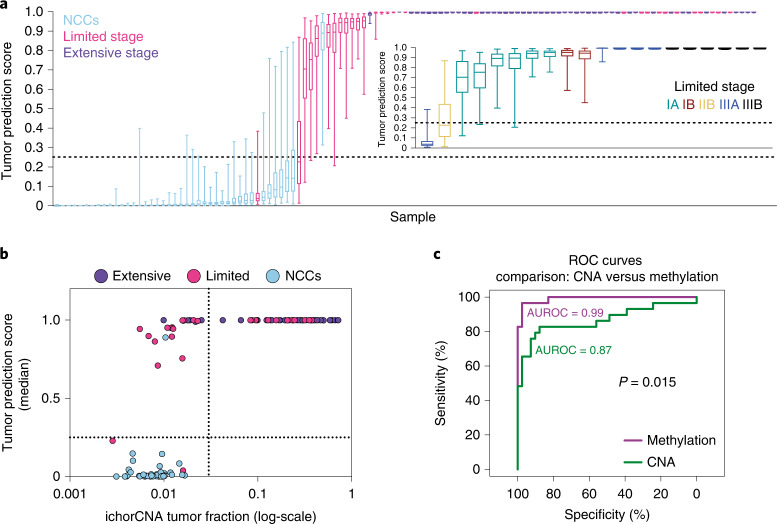
Methylation tumor prediction score applied to SCLC cfDNA samples. **a**, Box plots of classifier tumor prediction scores for 78 held-out SCLC cfDNA samples (29 limited stage and 49 extensive stage) and 41 held-out NCC cfDNA samples from applying the 100 classifiers trained on CDX/PDX synthetic spike-in samples. Boxes mark the 25th percentile (bottom), median (central bar) and 75th percentile (top); whiskers extend to minimum and maximum points. Dotted lines indicate the tumor prediction score cutoff value of 0.25. Inset plot shows tumor prediction scores for 20 out of 29 limited stage patients who had detailed staging information available. **b**, Scatter-plot showing median classifier tumor prediction scores against ichorCNA tumor fraction values (on a log scale) for the 78 SCLC and 41 NCC cfDNA samples. The classifier tumor prediction scores are correlated with ichorCNA tumor fraction (Spearman’s *ρ* = 0.72). Dotted lines are indicating the cutoff for both measures. **c**, ROC curves and AUROC scores generated by using ichorCNA tumor fraction (CNA, green line) or median classifier tumor prediction score (methylation, purple line) to classify LS-SCLC (*n* = 29) and NCC cfDNA (*n* = 41) samples. *P* value is from a comparison of the AUROC scores using a two-sided DeLong’s test. Source data

**Extended Data Fig. 2:**
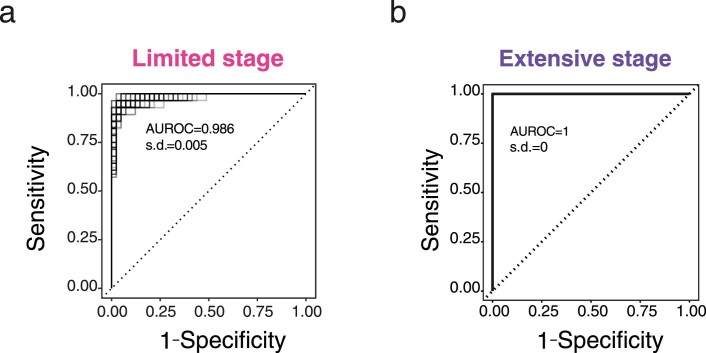
Sensitivity and specificity of the tumor/normal classifier. **a**,**b**, ROC curves from applying the 100 individual tumor/normal classifiers to 29 limited stage SCLC cfDNA samples and 41 NCC cfDNA samples (**a**), and to 49 extensive stage SCLC cfDNA samples and 41 NCC cfDNA samples (**b**). Source data

We next applied the trained tumor/healthy classifier to a validation set of 119 cfDNA samples, from NCCs not used in training (*n* = 41), patients with LS-SCLC (*n* = 29) and ES-SCLC (*n* = 49). The classifiers correctly assigned 93% and 100% of patients with LS-SCLC and ES-SCLC, respectively, with a statistically significant correlation of prediction scores with disease stage (Fig. 3a, inset; Kendall’s tau coefficient, 0.51; *P* = 0.0041). The performance in predicting SCLC yielded mean area under the receiver operating characteristic curve (AUROC) scores of 0.986 (s.d. = 0.005) and 1 (s.d. = 0), for LS-SCLC and ES-SCLC, respectively (Extended Data Fig. 2a,b). In contrast, although copy number-derived (ichorCNA) tumor fraction^16^ is correlated with classifier tumor prediction score (Spearman’s *ρ* = 0.72), it is less sensitive, detecting SCLC in 12 of 29 (41.4%) LS-SCLC and in 44 of 49 (89.82%) ES-SCLC (Fig. 3b). These data suggest that cfDNA methylation profiling substantially improves the sensitivity of SCLC detection, even in patients with early-stage, localized disease and low tumor burden (Fig. 3c).

### A prognostic cfDNA methylation score for SCLC

**Fig. 4:**
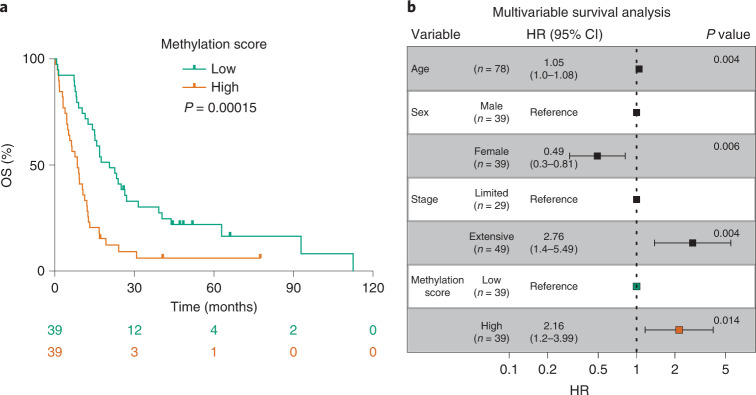
Methylation score predicts survival in patients with SCLC. **a**, Kaplan–Meier curves showing OS of the 78 patients with SCLC stratified by high and low methylation score (derived from cfDNA samples and calculated as the average *β*-value across 4,061 genomic regions used by the tumor/healthy classifier, then dichotomized using the median value). The number of patients at risk for each time point is indicated below the time point and color coded according to high or low groups. *P* value obtained by comparing the groups using a two-sided log-rank test. **b**, Forest plot showing the results of multivariable Cox proportional hazards regression modeling of OS for patients with methylation score high or low status. Error bars indicate 95% CI for the HR. *P* values were calculated using a two-sided Wald test. Source data

**Extended Data Fig. 3:**
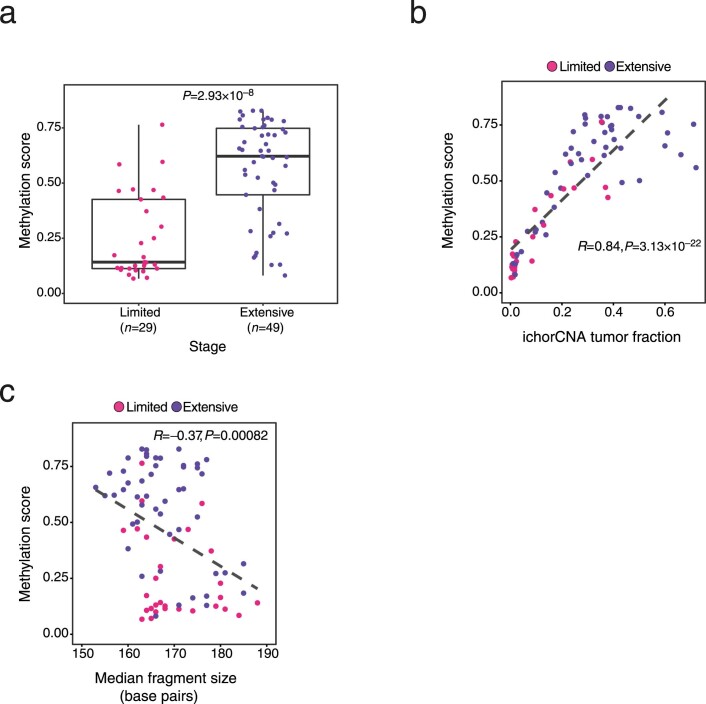
The methylation score as a surrogate of tumor burden. **a**, Box plot showing the methylation score (calculated as the average *β*-value across the 4,061 genomic regions used by the tumor/normal classifier) for cfDNA samples from limited or extensive stage patients. Boxes mark the 25th percentile (bottom), median (central bar) and 75th percentile (top). Whiskers extend to the most extreme value within 1.5-fold of interquartile range. Individual data points also shown. *P* value calculated by two-sided Mann-Whitney U test. **b**,**c** Scatter plots between the methylation score (as in **a**) and the copy-number estimated tumor fraction from ichorCNA (**b**), and median DNA fragment size (across the whole genome) from paired-end sequencing reads (**c**). Pearson correlation (*R* value) and two-sided *P* value are indicated. Black, dashed line shows linear regression fit. **a**–**c**, *n* = 78 cfDNA samples from independent SCLC patients (*n* = 29 limited stage and *n* = 49 extensive stage). Source data

We next hypothesized that measuring the level of tumor-specific methylation in each sample could be of clinical utility as a reflection of tumor burden. Therefore, we derived an SCLC methylation score for each cfDNA sample based on the average levels of methylation detected across the genomic regions used by the tumor/healthy classifier and performed an exploratory analysis to assess the prognostic utility of cfDNA methylation for overall survival (OS; Methods). This methylation score correlated positively with stage (Extended Data Fig. 3a; two-sided Mann–Whitney *U-*test, *P* < 0.0001) and ichorCNA tumor fraction (Extended Data Fig. 3b; Pearson correlation *R* = 0.84, two-sided *P* < 0.0001) and negatively with average DNA fragment size (Extended Data Fig. 3c; Pearson correlation *R* = −0.37, *P* = 0.00082), as expected for a surrogate of tumor burden. Kaplan–Meier analysis of the methylation score, dichotomized into low and high groups using the median, showed that patients with low scores had significantly longer OS than patients with high scores (Fig. 4a; median OS of 20.6 months and 8.5 months, respectively; two-sided log-rank test, *P* = 0.00015). The methylation score (continuous or dichotomized) was also significant in univariable Cox regression analysis, as was clinical stage (Supplementary Table 5). In multivariable Cox regression analysis, methylation score as continuous or dichotomized (Fig. 4b and Supplementary Table 6) remained significantly associated with OS in a model adjusting for age, sex and stage (hazard ratio (HR) = 3.60; 95% CI = 1.11–11.68; *P* = 0.033 for the continuous score). Compared to a model with only age, sex and stage, the models also containing methylation score had lower Akaike’s information criteria and Bayesian information criteria values and higher concordance index values. Overall, these data indicate that cfDNA methylation profiling has potential clinical utility in SCLC by allowing sensitive blood-based tumor detection and providing prognostic information beyond clinical stage; however, further work is needed with increased sample sizes and independent validation data to determine an optimal and robust cutoff.

### SCLC subtypes can be identified by cfDNA methylation

**Fig. 5:**
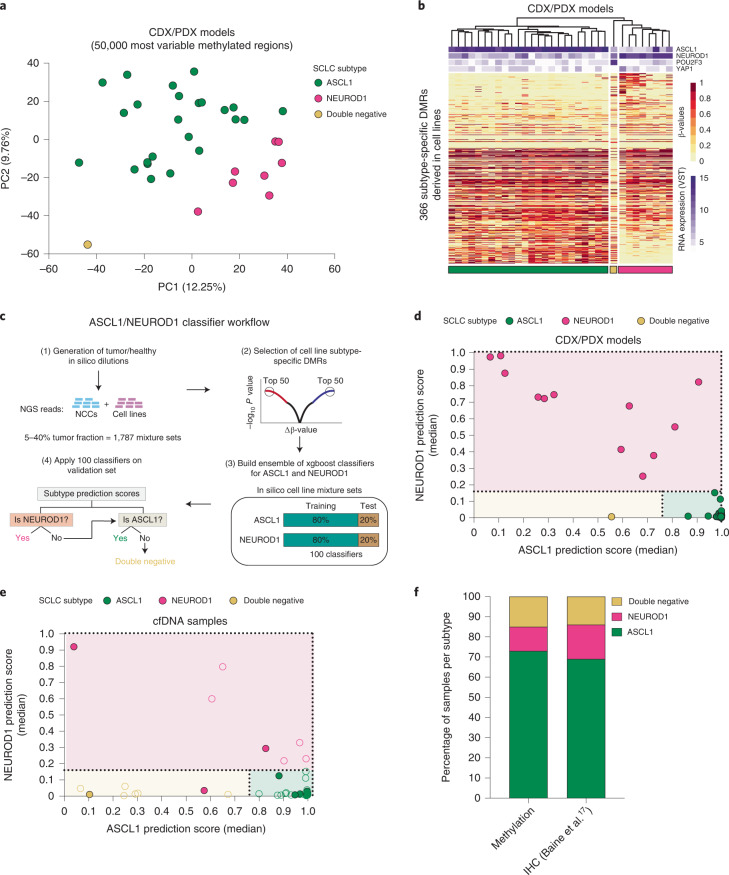
DNA methylation profiling identifies SCLC subtypes in both preclinical models and cfDNA samples. **a**, PCA plot of 33 CDX/PDX models (not including second models derived from the same patient), using *β*-values for the 50,000 most variable methylated regions across these models. CDX and PDX models segregated according to the expression of ASCL1, NEUROD1 (single or coexpressing with ASCL1) and POU2F3 (double negative) **b**, Hierarchical clustering heat map of *β*-values for 33 CDX/PDX models using 366 subtype-specific DMRs derived from publicly available DNA methylation data for 59 cell lines. Bars on the top show the expression values (variance-stabilizing transformation; VST) of ASCL1, NEUROD1, POU2F3 and YAP1 derived from RNA-seq data for each model. **c**, Analysis workflow for the generation of ASCL1 and NEUROD1 classifiers. **d**,**e**, ASCL1 and NEUROD1 classifier median prediction scores for 33 CDX/PDX models (**d**) and 56 cfDNA samples with an estimated tumor fraction of at least 4% (**e**). Color fill of dots indicates known subtype. In **e**, only cfDNA samples from patients who also generated a CDX model (*n* = 11) have known subtype. Dotted lines indicate classifier cutoff values. **f**, Bar plots of subtype distribution detected by cfDNA methylation (*n* = 56 patients) compared to subtype distribution detected by immunohistochemistry (IHC) of SCLC tissue samples (*n* = 159) from a previous study^17^. In **a**,**b**,**d** data for each CDX model are averaged over tumors from up to three independent mice. Source data

**Extended Data Fig. 4:**
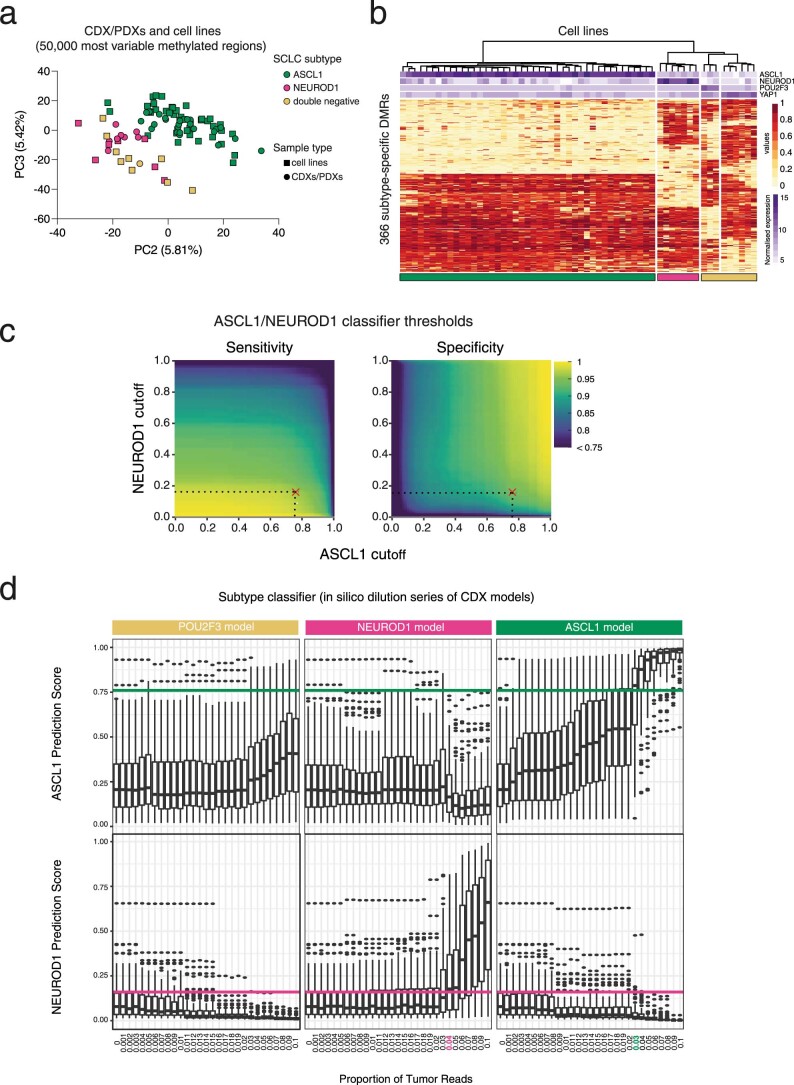
Identification of SCLC subtype-specific DMRs. **a**, PCA plot showing the 59 SCLC cell lines (43 ASCL1, 7 NEUROD1, 9 dual negative) and 33 CDX/PDX models (24 ASCL1, 8 NEUROD1, 1 dual negative; second models derived from the same patient were excluded), from PCA applied to *β*-values for the 50,000 most variable methylated regions according to the cell lines. *β*-values for each CDX model were averaged over up to three independent mice. **b**, Hierarchical clustering heatmap showing 366 subtype-specific DMRs derived by publicly available DNA methylation data from 59 cell lines. Bars on the top show the normalized expression values of ASCL1, NEUROD1, POU2F3 and YAP1 derived from Affymetrix Exon Microarrays for each cell line. **c**, Heatmaps showing sensitivity and specificity for varying cutoff values applied to the median prediction scores output by applying the ASCL1 and NEUROD1 classifiers to mixture sets in held-out test data (total of *n* = 1,787 mixture sets). Red crosses indicate the cutoffs (0.16 for NEUROD1; 0.76 for ASCL1) that jointly optimize the balanced accuracy metric (average of sensitivity and specificity) across both classifiers. **d**, Box plots of classifier prediction scores for *n* = 100 individual ASCL1 classifiers (top) or *n* = 100 individual NEUROD1 classifiers (bottom), applied to *in silico* serial dilutions of a POU2F3 (left), NEUROD1 (middle) or ASCL1 (right) CDX model mixed with an NCC cfDNA sample, with varying proportions of the CDX model in the mixture (*x*-axis). Boxes mark the 25th percentile (bottom), median (central bar) and 75th percentile (top). Whiskers extend to the most extreme value within 1.5-fold of interquartile range. Points lying outside the whiskers are plotted individually. Horizontal lines show the cutoffs for ASCL1 and NEUROD1 classifiers derived above. Source data

**Extended Data Fig. 5:**
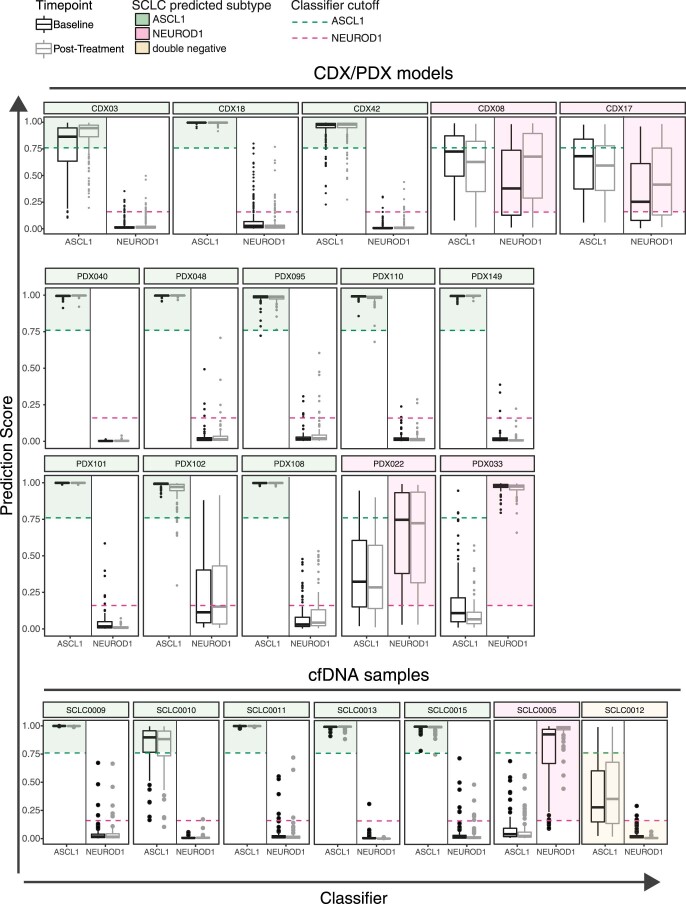
Prediction of SCLC subtype in post-treatment samples. Box plots showing prediction scores from *n* = 100 individual ASCL1 and NEUROD1 classifiers for a panel of paired CDX and PDX models, and paired cfDNA samples. CDX models were derived longitudinally from patients at baseline and post-treatment while paired PDX models were generated in vivo (as described in ref. ^30^). cfDNA samples were isolated from patients at baseline and again at post-treatment. Horizontal dotted lines show the median cutoffs for ASCL1 and NEUROD1 classifiers, 0.76 and 0.16 respectively. Colored regions indicate the predicted SCLC subtype. Boxes mark the 25th percentile (bottom), median (central bar) and 75th percentile (top). Whiskers extend to the most extreme value within 1.5-fold of interquartile range. Points lying outside the whiskers are plotted individually. Data for CDX models are averaged over tumors from up to three independent mice. Source data

We next sought to determine whether cfDNA methylation profiling could be used to subtype SCLC samples and recapitulate the molecular subtyping of our CDX/PDX models. Although numerous SCLC subtypes have been reported in the literature (achaete–scute complex homolog-like (ASCL1); neurogenic differentiation factor 1 (NEUROD1); atonal bHLH transcription factor 1 (ATOH1); POU class 2 homeobox 3 (POU2F3); Yes1 associated transcriptional regulator (YAP1); inflamed)^1,9^ a recent analysis of 174 SCLC tissue samples revealed the predominance of ASCL1, NEUROD1 and double-negative subtypes in clinical samples^17^. Therefore, we focused on classifying these three categories using methylation analysis. PCA applied to the top 50,000 most variable methylated regions in 33 CDX/PDX models with known molecular subtypes (RNA-seq) revealed accurate unsupervised segregation according to the three categories: NEUROD1 (high *NEUROD1* expression with or without coexpression of *ASCL1*, *n* = 8), ASCL1 (high *ASCL1* expression, *n* = 24) and double negative (low expression of *ASCL1* and *NEUROD1*, *n* = 1) (Fig. 5a) confirming methylation differences exist between SCLC subtypes. As we had only one example of the rarer double-negative subtype represented in our CDX/PDX model biobank, we utilized publicly available array methylation and expression data (National Cancer Institute Small Cell Lung Cancer Screening Project)^18^ from 59 previously characterized SCLC cell lines (43 ASCL1, 7 NEUROD1 and 9 double negative) as a training dataset to identify informative subtype-specific methylation. Initial work determined the feasibility of transforming array methylation data into normalized reads per million (nrpm) to build a subtype classifier applicable to our dataset. Good concordance was seen in CDX models processed through both platforms (Supplementary Table 7; Methods). Moreover, a joint PCA applied to the 59 cell lines together with 33 CDX/PDX models, using the top 50,000 most variable methylated regions according to the cell line samples only, showed concordance of the molecular subtypes identified independently in both datasets (Extended Data Fig. 4a). Using the transformed cell line array data, we identified 366 DMRs which discriminated between the three SCLC subtypes (Extended Data Fig. 4b and Supplementary Table 8). Clustering analysis of the 33 CDX/PDX models using the 366 subtype-specific DMRs found all models correctly clustered according to their transcriptional subtype (Fig. 5b). To build a cfDNA-based classifier, we applied a machine-learning approach that used the cell line-based subtype-specific DMRs and performed model training using in silico spike-ins of tumor reads derived from cell lines (5–40%) into NCC cfDNA samples (total of 1,787 mixture sets) (Fig. 5c). We analyzed sensitivity and specificity to derive optimal cutoffs to assign a sample as either NEUROD1, ASCL1 or double negative (Extended Data Fig. 4c; Methods). The validity of the classifiers was confirmed on CDX and PDX samples, which assigned all models correctly (Fig. 5d). To estimate the limit of detection of ASCL1 and NEUROD1 signal in cfDNA, we applied the classifiers to serial dilutions of CDXs representing the three categories and found positive signals for ASCL1 and NEUROD1 down to 3% and 4% tumor fraction, respectively (Extended Data Fig. 4d). Finally, we applied the classifiers to SCLC cfDNA samples with at least 4% tumor content (56 of 78), resulting in 10 of 11 samples with known subtypes (identified from a donor matched CDX model) correctly classified (Fig. 5e). Overall, 73% of the cfDNA samples were classified as ASCL1, 13% were classified as NEUROD1 and 14% were classified as being double negative, with the distribution of the subtypes correlating closely to previously published immunohistochemistry data from SCLC tissue samples (Fig. 5f; chi-squared test, *χ*^2^ = 0.628, d.f. = 2, *P* = 0.73). Next, we wanted to evaluate whether molecular subtyping of SCLC is feasible for longitudinal monitoring of the disease. We compared the prediction of the SCLC subtype in samples analyzed at baseline and after receiving chemotherapy (both CDX/PDX models and cfDNA) and found consistency of the predominant SCLC detected (Extended Data Fig. 5 and Supplementary Table 9). These data suggest that, with further evaluation in a larger cohort, cfDNA methylation profiling may provide a broadly applicable and accurate approach for molecular subtyping of patients with SCLC.

## Discussion

The minority of patients with SCLC who are eligible for surgery or chemoradiation with curative intent (approximately 30% of cases) achieve a 5-year survival rate of up to 65% (ref. ^19^); however, most patients present with advanced, incurable, metastatic disease. Minimally invasive biomarker assays are needed that enable earlier detection and monitoring of this deadly disease and that molecularly subtype SCLC (and inform dynamic subtype plasticity) to facilitate optimal stratification and scheduling of personalized therapies. Here we show that tumor-specific methylation patterns are readily detected in SCLC cfDNA samples, including in six of six patients with stage I tumors for which, with a parallel cfDNA assay, we failed to detect CNAs. We also show that the levels of tumor methylation detected in cfDNA correlated with survival outcomes. The high sensitivity of our approach opens up new avenues where cfDNA methylation profiling, alongside other technologies, could be included in large-scale lung cancer early-detection programs^20^ with potential for improved SCLC clinical outcomes and earlier detection of disease progression after chemotherapy, where further lines of treatment could be deployed sooner.

In what has been termed the ‘second golden age of SCLC research’^13^, the molecular subtyping of SCLC heralds new opportunities for stratified therapies. Several studies using cell line, engineered and patient-derived mouse models have shown differential therapeutic vulnerabilities across the SCLC molecular subtypes^9,21–23^. For instance, ASCL1-driven subtypes may be more susceptible to BCL2 apoptosis regulator and δ-like canonical Notch ligand 3 (DLL3) inhibitors, whereas NEUROD1-driven subtypes have been reported to be more sensitive to Aurora kinase inhibitors^24–26^. POU2F3-high cell lines are more resistant to chemotherapy compared to the other subtypes, but are sensitive to insulin like growth factor 1 receptor inhibition^21,27^. Clinical trials that enrolled patients with SCLC without molecular subtype stratification have been disappointing. Molecular profiling of SCLC tumors via a blood test could stratify patients and ultimately improve their clinical outcome.

This study shows that cfDNA methylation can identify molecular subtypes in SCLC, which warrants further validation in a larger independent patient cohort. A key advantage of blood-based molecular subtyping is circumventing the challenges often encountered in analyzing scant and often extensively necrotic tissue associated with SCLC tissue biopsies^11,28^. Methylation profiling also has the potential to bring insights into the biological behavior and clinical course of the different subtypes, including dynamic changes with disease progression.

We did not detect a switch of the predominant subtype after receiving treatment (Extended Data Fig. 5); however, the number of cfDNA samples analyzed is small (*n* = 7) and cannot exclude the presence of a subpopulation of cells with a different subtype emerging after treatment, which has been suggested in previous studies using single-cell RNA-seq^9,29^. In conclusion, circulating tumor DNA methylation may serve as a liquid biopsy to inform SCLC evolution, acquired resistance and future clinical trials of personalized treatment of patients with SCLC.

## Methods

### Ethical regulations

The research presented in this study complies with all the relevant ethical regulations. NCC samples were collected under the Community Lung Health Study (ethically approved study REC reference no. 17/LO415) or within the University of Manchester (University of Manchester ethics committee approval no. 2017-2761-4606) or purchased through Cambridge Bioscience (ethics committee approval no. 2019-7920-11797). Blood samples from patients with SCLC (ChemoRes trial) were collected after receipt of informed consent and according to ethically approved protocols: European Union CHEMORES FP6 contract no. LSHC-CT-2007-037665 (NHS Northwest 9 Research Ethical Committee). Blood samples from Memorial Sloan Kettering Cancer Center Institutional Review Board (IRB) protocol (IRB no.14-192A (4)) were collected after receipt of informed consent that met the requirements of the Code of Federal Regulations and the IRB/Privacy Board. Participants were not compensated. Additional double-spun plasma samples available through the National Cancer Institute Early Detection Research Network Funded Clinical Validation Center repository (IRB no. 000616) were shipped in dry ice from Vanderbilt Ingram Cancer Center to our institution.

### Blood samples collection from NCCs and patients with SCLC

Blood samples were collected in Cell-Free DNA BCT tubes (Streck), CellSave, BD Vacutainer K2 ethylenediaminetetraacetic acid (K2EDTA) for cfDNA analysis. Plasma was separated from whole blood by performing two sequential centrifugations (2,000*g*, 10 min) and stored at −80 °C before cfDNA analysis.

### Generation of CDX and PDX models

For CDXs, CTCs enriched from patients with SCLC were injected into the flank of an 8–16-week-old nonobese diabetic severe combined immunodeficient interleukin-2 receptor γ-deficient (NSG) mouse^7^. Tumors were collected once tumor volume reached 1,200 mm^3^; maximal tumor size was not exceeded. Female 8–16-week-old NSG mice were used to generate PDXs from primary tumors^30^. Tumor sizes were measured twice weekly and collected once tumor volume reached 2,000 mm^3^, maximal tumor size was not exceeded. All procedures were carried out in accordance with UK Home Office Regulations, the UK Coordinating Committee on Cancer Research guidelines and by approved protocols (Home Office Project license 40-3306/70-8252, Memorial Sloan Kettering Cancer Center Animal Care and Use Committee Protocol 04-03-009 and the Cancer Research UK Manchester Institute Animal Welfare and Ethical Review Advisory Body). In vivo studies were reported in accordance with ARRIVE guidelines 2.0. No new animal models were generated for this study.

### Genomic DNA extraction and fragmentation from preclinical models

Snap-frozen tumors from CDX and PDX models, generated as previously described^7,30,31^, were used to extract DNA. DNA was extracted by using Norgen Genomic DNA Isolation kit (catalog no. 24700) from up to three independent replicate tumors for CDX models and from up to two technical replicates for PDX models (Supplementary Table 1). Genomic DNA from healthy lung tissue was commercially bought (Origene). gDNA was quantified using NanoDrop Spectrophotometer (Thermo Scientific) and sheared to 200 bp (base pairs) on the Bioruptor Pico (Diagenode) followed by visualization on a 1.5% (w/v) agarose gel.

### Circulating cfDNA extraction and quantification

cfDNA was isolated by using the QIAmp MinElute ccfDNA MIDI kit (QIAGEN, catalog no. 55284) according to the manufacturer’s instructions and/or the QIAsymphony with the Circulating DNA kit (QIAGEN, catalog no. 1091063). Sheared gDNA obtained from preclinical models and healthy lung samples and cfDNA yields were quantified by using the TaqMan RNase P Detection Reagents kit (Life Technologies, catalog no. 4316831).

### T7-MBD-seq library preparation and NGS

Approximately 50 ng of sheared gDNA and between 1–35 ng of cfDNA were end-repaired and A-tailed (New England Biolabs, NEB, catalog no. E7595), dephosphorylated (FastAP Thermosensitive Alkaline Phosphatase, catalog no. EF0654) and ligated (Roche, catalog no. 07962355001) to custom oligonucleotides (Integrated DNA Technologies). These custom oligonucleotides consisted of a T7 RNA polymerase promoter sequence, Illumina read 1 sequencing primer-compatible sequence, a 10-bp sample barcode and a 6-bp unique molecular identifier (UMI), which had been pre-annealed to form a hairpin loop (patent PCT/GB2020/050635). The T7-MBD-seq library preparation method enabled sample multiplexing before methylation enrichment, which removed the need for filler DNA (as used by Huang et al.^32^) and enabled efficient analysis of samples at low cfDNA inputs (down to 1 ng). Ligated DNA was pooled (to a minimum of 75 ng in total) and combined with 0.3 ng of control methylated and 0.3 ng of control unmethylated *Arabidopsis thaliana* DNA (Diagenode, catalog no. C02040012). Ten percent of pooled ligated DNA was stored as input control, while the remaining 90% was subjected to methylation enrichment with the EpiMark Methylated DNA Enrichment kit (NEB, catalog no. E2600S) following the manufacturer’s instructions. The efficiency of the methylation enrichment was assessed by qPCR to detect the recovery of methylated (expected to be >20%) and unmethylated controls (expected to be <1%) in enriched samples (methylation capture; MeCap) relative to the input samples. Amplified RNA was then generated for both MeCap and input samples by in vitro transcription (IVT) using a complementary T7 promoter oligonucleotide and T7 RNA polymerase (NEB, catalog no. E2040S) following the manufacturer’s instructions. After IVT, a third of amplified RNA was subjected to single-strand ligation of an oligonucleotide adaptor containing an Illumina read 2 sequencing primer-compatible sequence (NEB, catalog no. M0373L) followed by reverse transcription (Thermo Scientific, catalog no. 18-090-050) and indexing PCR library amplification (Roche, catalog no. 07958897001). Libraries were paired-end sequenced on an Illumina NextSeq 500 or NovaSeq 6000.

### Read alignment

A nextflow^33^ (v.20.11.0) pipeline was generated to take the FASTQ files to analysis-ready quantitative sequencing enrichment analysis (QSEA) objects and is provided in the supplementary code. In this pipeline, FASTQ files were trimmed to all have the same initial length of 91 and 61 bp for R1s and R2s, respectively (including the 26-bp construct on R1), the UMI removed using umi-tools^34^ (v.1.0.1) and samples were demultiplexed and trimmed for adaptor sequences using cutadapt (10.14806/ej.17.1.200) (v.3.0). Reads were aligned to the GRCh38 reference genome using bwa mem (https://arxiv.org/abs/1303.3997) (v.0.7.17). Samples from mouse explants were also aligned to the mouse genome mm10 before using bamcmp^35^ (v.2.0) to remove those reads that align better to the mouse genome, using the alignment score metric. BAM files were deduplicated using umi-tools^34^ (v.1.0.1), using the start position of R1 and the UMI, ignoring the template length (fragment length), followed by running samtools^36^ (v.1.9) fixmate to assign mate quality scores.

### QSEA analysis

The QSEA R package^37^ (v.1.16) was used to analyze BAM files, with the use of a custom R package to extend QSEA (https://github.com/cruk-mi/mesa). The entire genome was tiled into 300-bp non-overlapping windows, with the removal of windows lying within the encode exclusion list regions^38^ (v2) and a further set of 3,753 windows with overrepresentation in our initial non-enriched input samples. Reads were then uniquely assigned into these 8,956,617 bins according to their midpoint location. Reads were filtered by keeping read pairs where either end of the pair mapped with a mapping quality (MAPQ) score of at least 10, or unpaired R1s with a MAPQ at least 10, using Rsamtools (v.2.6.0). For paired reads, a fragment length between 50–1,000 bp was required and for both paired and unpaired reads, a distance along the reference genome of at least 30 bp was required. Non-paired R1s were extended to the average of the length of the paired reads within that sample. Copy number variations were calculated from the non-enriched input sequencing for each sample, using HMMcopy^39^ (v.1.32) with base parameters over 1-Mbp windows. Each sample was normalized for library size using TMM (trimmed mean of M values, part of QSEA) with a pooled reference sample of eight NCCs. *β*-values (a scaled measure of methylation between 0 and 1) for each window in each sample were estimated within QSEA using the ‘blind calibration’ method^37^; windows with insufficient reads to estimate a *β*-value were returned as NAs.

### Ichor CNA

IchorCNA^16^ (v.0.3.2) was also used to give an estimate of the tumor fraction for each non-enriched input cfDNA sample, using a panel of normals generated from the NCC cfDNA samples, a 1-Mbp window size and without estimating subclonal populations. Estimated tumor fractions <0.03 were considered below the limit of detection, as in Adalsteinsson et al.^16^.

### Quality controls

FastQC (v.0.11.7), Qualimap^40^ (v.2.2) and Fastq-screen^41^ (v.0.14) were used for quality control of sequencing data, all visualized within MultiQC^42^ (v.1.9).

NGSCheckMate^43^ (v.1.0.0) was used to verify that all samples matched as expected in the tool output, including with previous RNA-seq data for the CDX and PDX samples, as well as the corresponding cfDNA from the same patients.

To calculate the relative enrichment scores, we followed the MEDIPS R package^44^ (v.1.42), calculating the total density of cytosine-guanine (CGs) contained within the mapped DNA positions (on the reference sequence) and dividing by the total density of CGs across the entire reference sequence. Samples with a relative enrichment <2.5 are excluded as being low quality.

Using a set of 805 windows that correspond to CpG sites that were shown to be always methylated in methylation array data from cancer and noncancer samples^45^, we required at least 40% of these windows to have a *β*-value of 0.8 or above.

### Differential methylation analysis

To calculate DMRs, we used the QSEA package, which implements a negative binomial generalized linear model, adjusting for the region CpG density. A minimum nrpm count >1 in at least one sample was required to consider a window for differential methylation and an FDR of 0.001 was applied. A difference between the average *β*-values for each class, Δ*β*, was calculated and a Δ*β* > 0.5 and 0.3 was used to identify the most significant DMRs in preclinical models and cfDNA samples, respectively. DMRs were annotated using the ChIPseeker R package^46^ and were mapped to CGIs, shores and shelves by using a list of CGIs (GRCh38) downloaded from Genome Browser^47^ annotation track database. CGIs were then extended by 2 kb using the plyranges R package^48^ (upstream and downstream) to identify shores and further 2 kb to identify shelves.

### Dilution series

To estimate the tumor fraction required to correctly call samples with each classifier, we generated an in silico dilution series using fastq-tools (v.0.8.3; https://github.com/dcjones/fastq-tools), mixing together raw, unfiltered reads between a cancer sample (H446 cell line or CDX) and a validation set NCC cfDNA at various proportions to make 20 million FASTQ read pairs, followed by our standard processing pipeline as detailed above. For the predictions, only those individual classifiers that had not been trained using the corresponding CDX (when relevant) were used.

### Tumor/healthy classifier

We split the NCC cfDNA samples into training and validation sets, with 38 NCC cfDNA samples used for training of the classifiers and 41 NCC samples held for the validation set. To train the classifier, we generated 1,951 synthetic mixture sets by mixing processed fragment counts between samples, either CDX/PDX samples with a NCC cfDNA at proportions between 0.5–5% or a mixture of two NCC samples, all at varying numbers of fragments.

A set of 4,061 SCLC-specific DMRs were identified that were differentially methylated between the CDX/PDX samples and both the healthy lung and the 38 training NCC cfDNA samples (both comparisons with a FDR of 0.001 and a Δ*β* ≥ 0.5).

An ensemble set of 100 classifiers was then built on these synthetic mixture sets and these windows, including mixtures built from 80% of the NCCs and 80% of the CDX/PDX samples in each individual classifier, using Extreme Gradient Boosting^49^ (xgboost R package, v.1.3.2.1) within the R tidymodels (v.0.1.3) framework, with default parameters (except trees, 500 and learn_rate, 0.02).

To derive a cutoff for the ensemble of classifiers from test data, we applied each of the classifiers to the remaining mixture sets that were not seen by that classifier during model training (together consisting of 20% of the NCC and 20% of the CDX/PDX samples). For each mixture set, we calculated the median of the 100 resulting prediction scores and compared against the ground truth (NCC mixture or SCLC mixture). We took the value of the cutoff that optimizes the balanced accuracy metric (the average of sensitivity and specificity). This cutoff was given by 0.25 with a balanced accuracy of 0.95 (0.93 sensitivity and 0.96 specificity).

The ensemble of trained classifiers was then applied to the remaining 41 held-out NCC cfDNA samples and all 78 SCLC cfDNA samples as a validation set, giving a median prediction score as well as showing the variability between classifiers. For cfDNA samples with an associated CDX model, only classifiers that did not use that CDX sample are used. The associated cutoff was also applied to the median predictions to give a hard assignment of each validation sample as either NCC or SCLC. Feature importance was estimated for each classifier using the vip R package (v.0.3.2; 10.32614/RJ-2020-013) and averaged over the ensemble (Supplementary Table 4).

### Methylation score and survival analysis

The 4,061 DMRs used within the tumor/healthy classifier were used to compute a ‘methylation score’, defined as the average of the *β*-values across these windows. Univariable Cox proportional hazards regression analysis for OS was performed for the following variables: methylation score (continuous), dichotomized methylation score (using median as a cutoff), age, sex and clinical stage. Additionally, for the categorical variables, Kaplan–Meier curve analysis and log-rank tests were carried out. The proportional hazards assumption was investigated using Schoenfeld residuals. Multivariable Cox proportional hazards regression models were fitted with methylation score as either a dichotomized or continuous variable, adjusting for age, sex and clinical stage. These models were compared to a model including only age, sex and clinical stage, using ﻿the Akaike information criterion, Bayesian information criterion and concordance index. Survival analysis was performed using the survival (https://CRAN.R-project.org/package=survival) and survminer (https://CRAN.R-project.org/package=survminer; R packages, v.3.2.11 and v.0.4.9, respectively).

### Assess the feasibility of using cell line array data to generate a SCLC subtype classifier

SCLC cell lines methylation data (from Illumina EPIC arrays^21^) and transcript data (from Affymetrix Exon Microarrays^18^) for SCLC cell lines were downloaded from sclccelllines.cancer.gov/sclc/downloads.xhtml (data time-stamped as December 2019) as pre-processed *β*-values and gene expression data. The expression levels of the genes *ASCL1*, *NEUROD1*, *POU2F3* and *YAP1* were used to assign subtypes to the cell lines (with a threshold of nine normalized expression for each gene), giving 43 *ASCL1*-, 7 *NEUROD1*-, 3 *POU2F3*- and 6 *YAP1*-expressing cell lines; excluding 7 that express both ASCL1 and NEUROD1. We termed the *POU2F3* and *YAP1*-expressing samples as dual negative, as they were expressing neither *ASCL1* nor *NEUROD1*.

### Converting methylation array data to QSEA objects

To use the array data to generate mixture sets as with the CDX/PDX data above, we developed a procedure to convert from *β*-values to estimates of nrpm. To do this, we used our pooled NCC reference (a mixture of eight cfDNA samples from healthy normal volunteers) within QSEA to generate a lookup table of counts to *β*-values, given the CG density for each window and for the enrichment/read depth of this pooled sample. We then applied this lookup table to estimate how many reads would have been captured by our T7-MBD-seq method in each window, given the *β*-values from the array, taking the maximum *β*-value where multiple probes lie within a window. Supplementary Table 7 shows the correlation between eight CDX samples sequenced using T7-MBD-seq and the estimated normalized reads per million from Infinium 450k array data of the same CDX models (restricted to the SCLC versus healthy lung DMRs), showing that matched samples have a high Spearman correlation.

### Unsupervised DNA methylation analysis for SCLC molecular subtyping

PCA was used to perform an unsupervised analysis of the *β*-methylation values for 33 CDX/PDX models (not including second models derived from the same patient), averaging across replicates. To take account of differences in enrichment between experimental runs, we carried out a batch-effect removal pre-processing step using the limma R package^50^ (v.3.46). *β*-values for each window were then centered and PCA was applied to the top 50,000 most variable windows, as determined by window s.d. across the 33 samples (Fig. 5a).

PCA was also applied to the 59 cell line samples, together with the CDX/PDX samples, by joining the *β*-values for the cell line samples with the (batch-corrected) *β*-values for the CDX/PDX samples. A further batch-effect removal step was then applied to account for systematic differences between the CDX/PDX and cell line data. The 50,000 windows with the highest s.d. in the cell line samples were used in the PCA (Extended Data Fig. 4a).

### Subtyping of CDX/PDX and corresponding cfDNA samples

For the CDX/PDX models, RNA-seq was processed as previously described^7,30,51^ followed by the calculation of variance-stabilized-transform values using the DESeq2 package^52^. Subtypes were assigned based on the highest TF expression among *ASCL1*, *NEUROD1* and *POU2F3* (dual negative), except for CDX38, which expresses high values of both *ASCL1* and *NEUROD1* and so was assigned as NEUROD1 positive. This gave 37 ASCL1 CDX/PDXs (from 24 patients, with 7 matched cfDNA samples), 12 NEUROD1 CDX/PDXs (from 8 patients, with 3 matched cfDNA samples) and 1 POU2F3 CDX (with a matched cfDNA sample).

### SCLC subtype classifier

Cell line DMRs were calculated between the subtypes (ASCL1, NEUROD1, YAP and POU2F3) using QSEA objects generated from arrays as detailed above, with an FDR rate of 0.001. These DMRs were ranked by their Δ*β*-values, with the 50 most hypermethylated and 50 most hypomethylated windows between a target class (ASCL1 or NEUROD1) and each of the other three subtypes used in the classifier for that target (300 windows in total). With windows being DMRs between multiple subtypes, this gave 261 distinct windows for the NEUROD1 classifier and 277 windows for ASCL1. Due to overlaps between these two sets of windows, this gave 366 windows in total.

Synthetic mixture sets were generated by mixing estimated read depths corresponding to the 59 cell line array *β*-values (as detailed above) with the same 38 NCC cfDNA samples as before, at concentrations between 5–40% from the arrays as well as varying numbers of reads, for a total of 1,787 mixtures. Two sets of 100 classifiers were generated using these mixture sets, one for predicting whether a sample is ASCL1 and one for predicting NEUROD1. Each classifier uses mixture sets corresponding to 80% of the NEUROD1 and dual-negative samples, with a similar number of ASCL1 samples (undersampling for class balance), as well as 80% of the NCCs in the same way as the tumor/healthy classifier to provide variability in exactly which mixtures were used in each classifier. Each classifier was trained using the R package xgboost^49^, v.1.3.2.1 with default parameters except trees, 500; learn_rate, 0.02.

We derived a cutoff for the NEUROD1 and ASCL1 ensemble classifiers in a similar way to the tumor/healthy classifier, using the mixture sets previously unseen during model training and calculating median prediction scores; however, here, the ASCL1 classifier cutoff was derived using only the samples that were not classified as NEUROD1 by the NEUROD1 classifier (the ASCL1 classifier cutoff was set after and was dependent on the NEUROD1 classifier cutoff). We considered a grid of cutoff values (with increments of 0.01) to jointly optimize the cutoffs for the two ensemble classifiers, using the average balanced accuracy across the two classifiers as the metric. This resulted in cutoffs of 0.16 for the NEUROD1 classifier and 0.76 for the ASCL1 classifier, with an optimal average balanced accuracy of 0.95 (NEUROD1 classifier, 0.95 balanced accuracy, 0.97 sensitivity and 0.94 specificity; and ASCL1 classifier, 0.95 balanced accuracy, 0.96 sensitivity and 0.94 specificity).

As a validation set, the classifiers were then applied to the CDX/PDX samples and the SCLC cfDNA samples (with a tumor fraction estimated by ichorCNA of at least 4% as suggested by our in silico dilutions) and hard predictions were made using the cutoffs derived on the mixture sets. Feature importance was estimated for each classifier using the vip package (v.0.3.2; 10.32614/RJ-2020-013) and averaged separately over the two ensemble classifiers (Supplementary Table 8).

### Statistics and reproducibility

Details of statistical analyses are provided throughout the text and in figure legends. All statistical tests were two-sided and, unless stated otherwise, results were considered significant at a *P* value threshold of 0.05. Multiple testing (FDR) correction was applied to *P* values arising from the DMR analysis. Most statistical tests used were nonparametric. For Pearson correlation hypothesis tests, data distributions were assumed to be normal but this was not formally tested. For Cox proportional hazards regression analysis, the proportional hazards assumption was investigated using Schoenfeld residuals. No statistical method was used to predetermine sample size but our sample sizes are similar to those reported in previous publications^53,54^. Samples were chosen and processed based on the availability of tissue and plasma samples at the time of data generation. Data failing quality controls or NCCs with a later known cancer diagnosis were excluded. The investigators were not blinded to the cancer status or subtype of any of the samples. As NCC cfDNA samples were required for both classifier training and validation, they were randomly allocated into two subsets, stratifying for the collection source. One subset was used for training the classifiers (within mixture sets) and the other was used to form part of the tumor/healthy classifier independent validation set (along with all the SCLC cfDNA samples). All other samples (CDX/PDX, cell lines and SCLC cfDNA) were only used either in classifier training or in the validation set. The majority of PDX models have two technical replicates (Supplementary Table 1). Read counts for these technical replicates were merged within QSEA to provide a single combined sample that was used for analysis. CDX models have up to three biological replicates (from different mice; Supplementary Table 1); these were kept as separate entries or were averaged, as indicated in the text or figure legends. Plots were generated with GraphPad Prism (v.9.2) and R (v.4.0.3), using ggplot2 (v.3.3.5) and pheatmap (v.1.0.12).

### Reporting summary

Further information on research design is available in the Nature Research Reporting Summary linked to this article.

## Supplementary information

Reporting Summary

Supplementary Tables Supplementary Tables 1–9.

## Data Availability

T7-MBD-seq data and shallow WGS data that support the findings of this study have been deposited in the European Genome–Phenome Archive under accession no. EGAS00001005739. Processed QSEA R objects are deposited in Zenodo at 10.5281/zenodo.5569261. Previously published array methylation and expression data that were reanalyzed here are available under GSE145156 and GSE73160. Previously published RNA-seq data from the CDXs and PDXs studied here are available from ArrayExpress under accession code E-MTAB-8465 (CDXs) and the database of Genotypes and Phenotypes under accession no. phs001249.v1.p1 (PDXs). Source data have been provided as Source Data files. All other data supporting the findings of this study are available from the corresponding author on reasonable request. Source data are provided with this paper.

## Source data

Source Data Fig. 1 Statistical Source Data.
Source Data Fig. 2 Statistical Source Data.
Source Data Fig. 3 Statistical Source Data.
Source Data Fig. 4 Statistical Source Data.
Source Data Fig. 5 Statistical Source Data.
Source Data Extended Data Fig. 1 Statistical Source Data.
Source Data Extended Data Fig. 2 Statistical Source Data.
Source Data Extended Data Fig. 3 Statistical Source Data.
Source Data Extended Data Fig. 4 Statistical Source Data.
Source Data Extended Data Fig. 5 Statistical Source Data.

## Code availability

Scripts for the analysis used in this paper are available at gitlab.com/cruk-mi/sclc-cfDNA-methylome-profiling and the R package is available at github.com/cruk-mi/mesa.
