# Supplementary material for: cfDNA methylome profiling for detection and subtyping of small cell lung cancers
Source: Nat Cancer. 2022 Aug 8;3(10):1260–70. doi: 10.1038/s43018-022-00415-9 (PMC9586870; doi:10.1038/s43018-022-00415-9)
Supplement: Supplementary file 1 — Reporting Summary [file 43018_2022_415_MOESM1_ESM.pdf]

nature portfolio

Corresponding author(s): Caroline Dive, Charles M. Rudin,Dominic G. Rothwell

Last updated by author(s): Jun 6, 2022

Reporting Summary

Nature Portfolio wishes to improve the reproducibility of the work that we publish. This form provides structure for consistency and transparency in reporting. For further information on Nature Portfolio policies, see our Editorial Policies and the Editorial Policy Checklist.

Statistics

For all statistical analyses, confirm that the following items are present in the figure legend, table legend, main text, or Methods section.

n/a	Confirmed	
☐	☒	The exact sample size (*n*) for each experimental group/condition, given as a discrete number and unit of measurement
☐	☒	A statement on whether measurements were taken from distinct samples or whether the same sample was measured repeatedly
☐	☒	The statistical test(s) used AND whether they are one- or two-sided<br>*Only common tests should be described solely by name; describe more complex techniques in the Methods section.*
☐	☒	A description of all covariates tested
☐	☒	A description of any assumptions or corrections, such as tests of normality and adjustment for multiple comparisons
☐	☒	A full description of the statistical parameters including central tendency (e.g. means) or other basic estimates (e.g. regression coefficient) AND variation (e.g. standard deviation) or associated estimates of uncertainty (e.g. confidence intervals)
☐	☒	For null hypothesis testing, the test statistic (e.g. *F*, *t*, *r*) with confidence intervals, effect sizes, degrees of freedom and *P* value noted<br>*Give P values as exact values whenever suitable.*
☒	☐	For Bayesian analysis, information on the choice of priors and Markov chain Monte Carlo settings
☒	☐	For hierarchical and complex designs, identification of the appropriate level for tests and full reporting of outcomes
☐	☒	Estimates of effect sizes (e.g. Cohen's *d*, Pearson's *r*), indicating how they were calculated

Our web collection on statistics for biologists contains articles on many of the points above.

Software and code

Policy information about availability of computer code

Data collection

No software was used for data collection.

Data analysis

Custom R scripts for the analysis presented in this paper are available at gitlab.com/cruk-mi/sclc-cfDNA-methylome-profiling.

The following tools were used:
BWA (0.7.17)
samtools (v1.9)
umi-tools (v1.0.1)
fastQC (v.0.11.7)
qualimap (v2.2)
bamcmp (v2.0)
multiQC (v1.9)
fastqscreen (v0.14)
cutadapt (v3.0)
NGSCheckmate (v1.0.0)
R (v4.0.3)
RStudioWorkbench (v1.4.1717-3)
NextFlow (v20.11.0)
fastq-tools (v0.8.3)

R packages:

BiocParallel (v1.24.1)

broom (v0.7.9)
BSgenome.Hsapiens.NCBI.GRCh38 (v1.3.1000)
ChIPpeakAnno (v3.24.2)
ChIPseeker (v1.26.2)
DESeq2 (v1.30.1)
devtools (v2.4.2)
furrr (v0.2.2)
GGally (v2.1.2)
ggplot2 (v3.3.5)
ggpubr (v0.4.0)
glue (v1.4.2)
hmmcopy (v1.32)
ichorCNA (v0.3.2)
janitor (v2.1.0)
kableExtra (v1.3.4)
limma (v3.46.0)
MEDIPS (v1.42)
org.Hs.eg.db (v3.12.0)
patchwork (v1.1.1)
pheatmap (v1.0.12)
plotly (v4.9.3)
plyranges (v1.10)
pROC (v1.17.0.1)
qsea (v1.16.0)
RColorBrewer (v1.1-2)
readxl (v1.3.1)
rmarkdown (v2.8)
Rsamtools (v2.6.0)
survival (v3.2.11)
survminer (v0.4.9)
tidymodels (v0.1.3)
tidyverse (v1.3.1)
vip (v0.3.2)
workflows (v0.2.2)
xgboost (v1.3.2.1)

For manuscripts utilizing custom algorithms or software that are central to the research but not yet described in published literature, software must be made available to editors and reviewers. We strongly encourage code deposition in a community repository (e.g. GitHub). See the Nature Portfolio guidelines for submitting code & software for further information.

Data

Policy information about availability of data

All manuscripts must include a data availability statement. This statement should provide the following information, where applicable:

- Accession codes, unique identifiers, or web links for publicly available datasets
- A description of any restrictions on data availability
- For clinical datasets or third party data, please ensure that the statement adheres to our policy

T7-MBD-seq data and shallow whole genome sequencing data that support the findings of this study have been deposited in the European Genome-Phenome Archive (EGA) under the accession number EGAS00001005739. Processed qsea R objects are deposited in Zenodo under the DOI 10.5281/zenodo.5569261. Previously published array methylation and expression data that were reanalysed here are available under GSE145156 and GSE73160. Previously published RNAseq data from the CDXs and PDXs studied here are available from ArrayExpress under accession code E-MTAB-8465 (CDXs) and the database of Genotypes and Phenotypes (dbGaP) under accession number phs001249.v1.p1 (PDXs).

Field-specific reporting

Please select the one below that is the best fit for your research. If you are not sure, read the appropriate sections before making your selection.

☒ Life sciences ☐ Behavioural & social sciences ☐ Ecological, evolutionary & environmental sciences

For a reference copy of the document with all sections, see nature.com/documents/nr-reporting-summary-flat.pdf

Life sciences study design

All studies must disclose on these points even when the disclosure is negative.

Sample size

No statistical methods were used to predetermine sample size. All CDX/PDX models with available tissue samples were profiled through T7-MBDseq. All SCLC cases with plasma samples available at the time of data generation were used. Non-cancer-control samples collected under the Community Lung Health Study were selected if deemed to be cancer-negative by CT scan performed at the time of blood draw.

Data exclusions

T7-MBDseq samples were excluded if they met any of the following criteria:
1. Relative methylation enrichment score (RelH) of less than 2.5.

2. Less than 40% of 805 hyperstable methylated regions have a beta value of 0.8 or above.
3. For NCC samples collected through the Community Lung Health Study, a known later cancer diagnosis was given.

Cell lines which expressed both ASCL1 and NEUROD1 were excluded from analysis.

Replication	Where possible, three independent replicate tumours for CDX models and two technical replicates for PDX models were used. Methylation profiles were found to be concordant between replicates and also correlated with previously described methylation patterns in SCLC tissue. We also found agreement between methylation patterns in tissue and in cfDNA.<br>Classifiers were trained on synthetic mixture sets of CDX/PDX samples and a subset of non-cancer controls (tumour/normal classifiers), or cell lines and a subset of non-cancer controls (ASCL1/NEUROD1 classifiers) . To guard against overfitting, cut-offs associated to each classifier were derived by applying each of the classifiers to the remaining mixture sets which were not seen by that classifier during model training (together comprising 20% of the NCC and 20% of the CDX/PDX samples or cell lines). Ensembles of 100 tumour/normal classifiers and 100 ASCL1/NEUROD1 classifiers were trained to ensure robustness and reproducibility. CfDNA samples from non-cancer controls (the subset not used in training) and from cancer individuals were used as an independent validation set for the tumour/normal classifier. The cfDNA samples from cancer individuals were also used for the independent validation set for the ASCL1/NEUROD1 classifier, together with the CDX/PDX models, since these were not used for training of the ASCL1/NEUROD1 classifiers.<br>The performance of both classifiers were not replicated in additional cfDNA samples since the aim of this study was to assess the feasibility of using cfDNA methylation profiling for detection and subtyping of SCLC. A further validation in a larger independent patient cohort will be performed.
Randomization	For classifier development, samples were randomized where necessary to ensure no overlap between training, test and validation sets. Importantly, since non-cancer control cfDNA samples were required for both classifier training and validation, they were randomly allocated into two subsets, stratifying for the collection source. One subset was used for training the classifiers (within mixture sets) and the other was used to form part of the tumour/normal classifier validation set (along with all the SCLC cfDNA samples). All other samples (CDX/PDX, cell lines and SCLC cfDNA) were only used either in classifier training or in the validation set.
Blinding	Due to the exploratory nature of this study, aiming to assess the feasibility of using cfDNA methylation profiling for detection and subtyping of SCLC, the investigators were not blinded to the cancer status or subtype of any of the samples.

Reporting for specific materials, systems and methods

We require information from authors about some types of materials, experimental systems and methods used in many studies. Here, indicate whether each material, system or method listed is relevant to your study. If you are not sure if a list item applies to your research, read the appropriate section before selecting a response.

Materials & experimental systems

n/a	Involved in the study
☒	☐ Antibodies
☒	☐ Eukaryotic cell lines
☒	☐ Palaeontology and archaeology
☐	☒ Animals and other organisms
☐	☒ Human research participants
☒	☐ Clinical data
☒	☐ Dual use research of concern

Methods

n/a	Involved in the study
☒	☐ ChIP-seq
☒	☐ Flow cytometry
☒	☐ MRI-based neuroimaging

Animals and other organisms

Policy information about studies involving animals; ARRIVE guidelines recommended for reporting animal research

Laboratory animals	For CDXs, CTCs enriched from patients with SCLC were injected into the flank of a 8–16week old nonobese diabetic (NOD) severe combined immunodeficient (SCID) interleukin-2 receptor γ–deficient (NSG) mouse 7. Tumours were harvested once tumour volume reached 1,200 mm3, maximal tumour size was not exceeded. Female 8-16 week old NSG mice were used to generate PDXs from primary tumours 30. Tumour sizes were measured twice weekly and harvested once tumour volume reached 2,000 mm3, maximal tumour size was not exceeded. All procedures were carried out in accordance with Home Office Regulations (UK), the UK Coordinating Committee on Cancer Research guidelines and by approved protocols (Home Office Project license 40-3306/70-8252, Memorial Sloan Kettering Cancer Center Animal Care and Use Committee Protocol 04-03-009 and the Cancer Research UK Manchester Institute Animal Welfare and Ethical Review Advisory Body). In vivo studies have been reported in accordance with ARRIVE Guidelines 2.0. No new animal models were generated for this study.
Wild animals	This study did not involve wild animals.
Field-collected samples	This study did not involve field-collected samples.
Ethics oversight	As stated above, all procedures were carried out in accordance with Home Office Regulations (UK), the UK Coordinating Committee on Cancer Research guidelines and by approved protocols (Home Office Project license 40-3306/70-8252 and Cancer Research UK Manchester Institute Animal Welfare and Ethical Review Advisory Board).

Note that full information on the approval of the study protocol must also be provided in the manuscript.

Human research participants

Policy information about studies involving human research participants

Population characteristics	The patient data relating to this study can be found Extended Data table 1-3 which include sex, age,stage and smoking history. The SCLC cfDNA cohort comprises 38 male and 40 female patients with a median age of 67. 29 and 49 were limited and extensive stage patients, respectively. The non-cancer control cfDNA cohort comprises 39 male and 40 female individuals with a median age of 63.
Recruitment	Patients with a diagnosis of SCLC were recruited by physician referral according to ethically approved protocols shown below. Non-cancer controls were locally collected (n=8), commercially bought from Cambridge Biosciences (n=26) or part of the Community Lung Health Study trial (n=45) as detailed in the methods.<br><br>Selection bias may have been introduced due to the availability of patient samples, and the requirement of sufficient cfDNA in a sample to pass our quality control steps may lead to a bias towards patients with more severe disease. Limited stage SCLC samples were predominantly collected at Vanderbilt Ingram Cancer Center, which may have caused a bias due to differing underlying populations. More extensive stage than limited stage samples were collected, as is typical for diagnosis of SCLC. To aid interpretation, we have presented results separately for extensive stage and limited stage where appropriate.<br><br>Our non-cancer control cfDNA samples are predominantly risk- and age-matched (45 samples vs 26 age-matched only samples and 8 unmatched samples). However, we see no evidence of bias in the tumour/normal classifier performance; we present results for each sample separately and find similar performance on all three groups.
Ethics oversight	Non-cancer-control samples were collected under the Community Lung Health Study (ethically approved study REC reference: 17/LO415) or within the University of Manchester (University of Manchester ethics committee approval no. 2017-2761-4606) or purchased through Cambridge Bioscience (ethics committee approval no. 2019-7920-11797). Blood samples from patients with SCLC (ChemoRes trial) were collected after receipt of informed consent and according to ethically approved protocols: European Union CHEMORES FP6 Contract number LSHC-CT-2007-037665 (NHS Northwest 9 Research Ethical Committee). Blood samples from Memorial Sloan-Kettering Cancer Center (MSKCC) IRB protocol (IRB#:14-192 A (4)) were collected after receipt of informed consent that met the requirements of the Code of Federal Regulations and the Institutional Review Board/Privacy Board.

Note that full information on the approval of the study protocol must also be provided in the manuscript.
